# Inelastic Neutron Scattering Study of Phonon Density of States of Iodine Oxides and First-Principles Calculations

**DOI:** 10.1021/acs.jpclett.3c02357

**Published:** 2023-11-02

**Authors:** Alexander I. Kolesnikov, Aravind Krishnamoorthy, Ken-ichi Nomura, Zhongqing Wu, Douglas L. Abernathy, Ashfia Huq, Garrett E. Granroth, Karl O. Christe, Ralf Haiges, Rajiv K. Kalia, Aiichiro Nakano, Priya Vashishta

**Affiliations:** † Neutron Scattering Division, Oak Ridge National Laboratory, Oak Ridge, Tennessee 37831-6473, United States; ‡ J. Mike Walker ’66 Department of Mechanical Engineering, Texas A&M University, College Station, Texas 77843, United States; § Collaboratory for Advanced Computing and Simulations, Department of Chemical Engineering & Materials Science, Department of Physics & Astronomy, and Department of Computer Science, University of Southern California, Los Angeles, California 90089-0242, United States; ∥ School of Earth and Space Sciences, University of Science and Technology of China, Hefei, Anhui 230026, China; ⊥ Sandia National Laboratories, Livermore, California 94551, United States; # Loker Research Institute and Department of Chemistry, University of Southern California, Los Angeles, California 90089-1661, United States

## Abstract

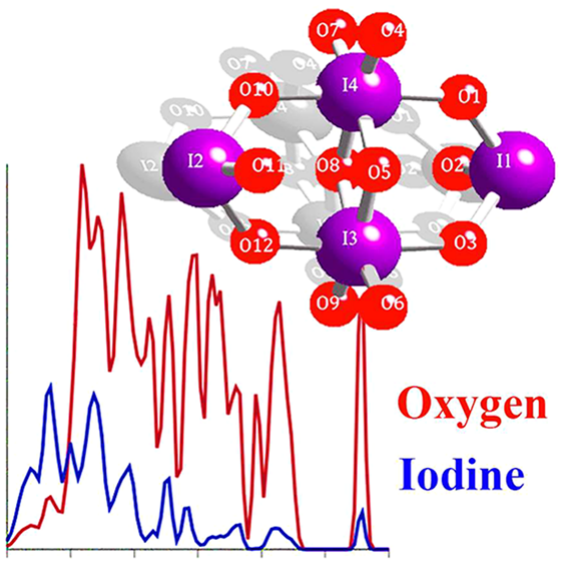

Iodine oxides I_2_O_*y*_ (*y* = 4, 5, 6) crystallize into atypical structures that fall between molecular- and framework-base types and exhibit high reactivity in an ambient environment, a property highly desired in the so-called “agent defeat materials”. Inelastic neutron scattering experiments were performed to determine the phonon density of states of the newly synthesized I_2_O_5_ and I_2_O_6_ samples. First-principles calculations were carried out for I_2_O_4_, I_2_O_5_, and I_2_O_6_ to predict their thermodynamic properties and phonon density of states. Comparison of the INS data with the Raman and infrared measurements as well as the first-principles calculations sheds light on their distinctive, anisotropic thermomechanical properties.

Iodine oxides (IO) play a critical role in many important physiochemical phenomena and, once their basic thermodynamic and chemical properties are understood, may lend themselves to a series of overarching technological applications.^1,2^ IO generated by photolysis of biogenic iodocarbons emitted from marine algae, interact with O_3_ under ultraviolet radiation to produce aerosol and cloud condensation nuclei, hence contributing to climate change.^3−8^ IO are also versatile and effective oxidants. I_2_O_5_ is known to oxidize alcohol, the nine-membered amide and cycloalkan[*b*]indoles, whereby useful products can be synthesized in atom-efficient, chemo-selective, and environmentally friendly ways.^9,10^ Furthermore, I_2_O_6_ and I_2_O_7_ are predicted to be effective neutralizing reagents that defeat the functionality of chemical and biological agents.

Notwithstanding a long history of research, the experimental characterization of IO has been limited mainly to I_2_O_4_, I_2_O_5_, and a few intermediate compounds associated with interactions with water and sulfuric acid. First-principles calculations have been performed in various molecular IO, INO_3_, and HOI species,^11,12^ with only one prior calculation of the structural and thermodynamic properties of bulk I_2_O_4_, I_2_O_5_, and I_2_O_6_ reported in the literature.^13^ Among the I_2_O_*y*_ (*y* = 4–7) materials, the crystal structures of the *y* = 4, 5, and 6 members are found to conform to the trend of changing from polymeric-like to framework-like structures with increasing oxygen content. Consequently, the thermodynamic properties of I_2_O_6_, particularly under high-pressure and high-temperature conditions, are expected to differ from those of I_2_O_4_ and I_2_O_5_. To this end, the properties of I_2_O_6_ are of most interest, but the limited chemical stability of this material has impeded the synthesis of bulk I_2_O_6_, as well as its experimental characterization.

Important strides in materials science have been made by utilizing both theoretical modeling and experimental characterization, particularly based on neutron and X-ray scattering techniques.^14−19^ In this paper, we present experimental and theoretical studies combining inelastic neutron scattering and spectroscopic measurements with first-principles calculation of the structure, phonon density of states (PDOS), and thermodynamic properties of bulk anhydrous I_2_O_*y*_ (*y* = 4–6). Our theoretical results are compared with the experimental neutron data. After calibrating both the structure and lattice dynamics, we carried out calculations to predict the equations of states (EOS) and other thermodynamic properties of the three IOs.

## Synthesis of I_2_O_6_ and I_2_O_5_

The previously described literature methods for the synthesis of I_2_O_6_ have serious drawbacks. The thermal decomposition of H_5_IO_6_ in a vacuum^20^ is difficult to control and produces I_2_O_6_ contaminated with side products. Furthermore, its preparation was applicable only to very small amounts of I_2_O_6_. While the dehydration of H_5_IO_6_ in concentrated H_2_SO_4_ at 70 °C was reported to proceed only very slowly during the course of one month,^21^ dehydration of a mixture of H_5_IO_6_ and HIO_3_ with 65% oleum resulted in impurities of (IO_2_)_2_S_2_O_7_.^22^ During the course of our study, it was found that the dehydration of a mixture of H_5_IO_6_ and HIO_3_ with a stoichiometric amount of SO_3_ in 100% sulfuric acid at elevated temperature yields pure I_2_O_6_ on a several-gram scale within a few hours:
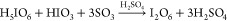
1An excess of SO_3_ should be avoided because it results in the formation of (IO_2_)_2_S_2_O_7_. The sulfuric acid can be removed by washing with anhydrous trifluoroacetic acid under anhydrous conditions.

During this study, it was found that commercially available samples of I_2_O_5_ were not suitable for neutron scattering experiments, because they contained small amounts of hydrogen-containing compounds. These impurities could not be removed by heating the samples to 200 °C in a vacuum for 36 h. Pure I_2_O_5_ suitable for neutron experiments was then prepared via the thermal decomposition of I_2_O_6_:
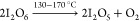
2This is an exothermic reaction, and care must be taken to remove the heat of reaction, especially for large reaction batches. Otherwise, the reaction mixture can overheat, which results in the thermal decomposition of I_2_O_5_ to I_2_. We observed such an event when a 250-mL glass flask loaded with 10 mmol of I_2_O_6_ heated to 145 °C in an oil bath resulted in a run-away exothermic decomposition reaction and partial decomposition of I_2_O_5_ to I_2_.

All reactions were carried out under dry nitrogen, using standard Schlenk techniques. Nonvolatile materials were handled in the dry nitrogen atmosphere of a glovebox. Glassware was heated out under a vacuum before use. Raman spectra were recorded directly in the Teflon reactors in the range of 4000–80 cm^–1^ on a Bruker Equinox 55 FT-RA spectrophotometer, using a Nd:YAG laser at 1064 nm with power levels of 200 mW. IR spectra were recorded in the range of 4000–400 cm^–1^ on a Midac, M Series, FT-IR spectrometer, using KBr pellets. The pellets were prepared inside the glovebox using an Econo mini-press (Barnes Engineering Co.) and transferred inside a closed container to the spectrometer before placing them quickly into the sample compartment, which was purged with dry nitrogen to minimize exposure to atmospheric moisture and potential hydrolysis of the sample. The starting materials HIO_3_, H_5_IO_6_, H_2_SO_4_, and oleum (all Aldrich) were used without further purification. Trifluoroacetic acid (SynQuest Laboratories, Alachua, FL) was freshly distilled from P_2_O_5_ prior to use. I_2_O_5_ was prepared by thermal decomposition of I_2_O_6_.^21,22^ The crystal structure and purity of the I_2_O_6_ and I_2_O_5_ samples were checked by neutron diffraction and Raman, IR, and neutron spectroscopies.

### Preparation of Diiodine Oxide, I_2_O_6_

Finely ground HIO_3_ (100.41 g, 0.571 mol) and H_5_IO_6_ (130.10 g, 0.571 mol) were loaded into a Schlenk flask, and concentrated sulfuric acid (400 mL) was added. The mixture was heated to 90 °C and stirred. After ∼0.4 h, a yellow suspension was formed. The flask was removed from the heat source and 30% oleum (184 mL) was added under vigorous stirring to the hot suspension. The mixture was stirred at ambient temperature for 16 h. The mixture was filtered through a fine porcelain filter frit, and the yellow solid was washed 10 times with 200 mL of dry trifluoroacetic acid. The solid was then dried under vacuum at ambient temperature for 14 h. We thus obtained 197.2 g of finely powdered, yellow I_2_O_6_ (the weight expected for 0.571 mol I_2_O_6_ is 199.7 g).

### Preparation of Diiodine Oxide, I_2_O_5_

I_2_O_6_ (2.50 g, 7.15 mmol) was loaded into a glass ampule that was equipped with a grease-free Kontes HiVac valve and a Teflon stopcock. After the ampule was evacuated, the valve was closed and the vessel was placed in an oil bath at ambient temperature. The bath was heated to 130 °C. After 0.5 h, the temperature was raised to 150 °C. After another 0.5 h, the temperature was raised to 160 °C and after another 0.5 h, to 170 °C. The solid was kept at this temperature for 12 h and, subsequently, it turned completely colorless. The ampule was allowed to cool to ambient temperature and connected to a glass vacuum line. The amount of noncondensible gas produced inside the ampule was determined by pressure, volume, and temperature measurements to be 7.2 mmol. We thus obtained 2.34 g of finely powdered, colorless I_2_O_5_ (the weight expected for 7.15 mmol of I_2_O_5_ is 2.39 g).

## Neutron Scattering Experiments

The crystal structures of anhydrous I_2_O_5_ and I_2_O_6_ over the temperature range of 4–300 K were investigated by neutron powder diffraction, using the POWGEN diffractometer at the Spallation Neutron Source (SNS) of Oak Ridge National Laboratory.^23^ Inelastic neutron scattering (INS) was carried out at low temperature (10 K) using the chopper spectrometer, ARCS, which was also located at SNS.^24^ To achieve the best energy resolution in the whole vibrational spectra of I_2_O_*y*_, Δ*E*/*E*_*i*_ ≈ 1%–3%, we used the incident energies of *E*_*i*_ = 20, 45, 85, and 150 meV, selected by the Fermi chopper. I and O atoms mainly scatter neutrons coherently; therefore, neutron scattering had to be averaged over a large range of neutron momentum transfer in order to obtain the vibrational density of states of polycrystalline samples. The quality of the average was determined by the ratio *R* of the volume of the reciprocal space covered in the INS experiments to the volume of the Brillouin zone of the crystal.^25^ The INS data were recorded over a wide range of scattering angles: 28°–135° in the horizontal plane and ±26° in the vertical directions, providing large coverage of inverse space, and hence resulting in large *R* values (>10^3^). Thus, the condition of averaging was fulfilled in the whole range of energy transfers (2–150 meV).

The samples were sealed inside a helium-filled container throughout the experiments to avoid an interaction with the ambient atmosphere. The extraordinarily large neutron incoherent scattering cross section from hydrogen, during a long run, served as a sensitive test of the presence of water or H-containing impurities. We found no evidence of an incoherent-scattering background or local-mode vibrations due to hydrogen species in the samples.

**Figure 1:**
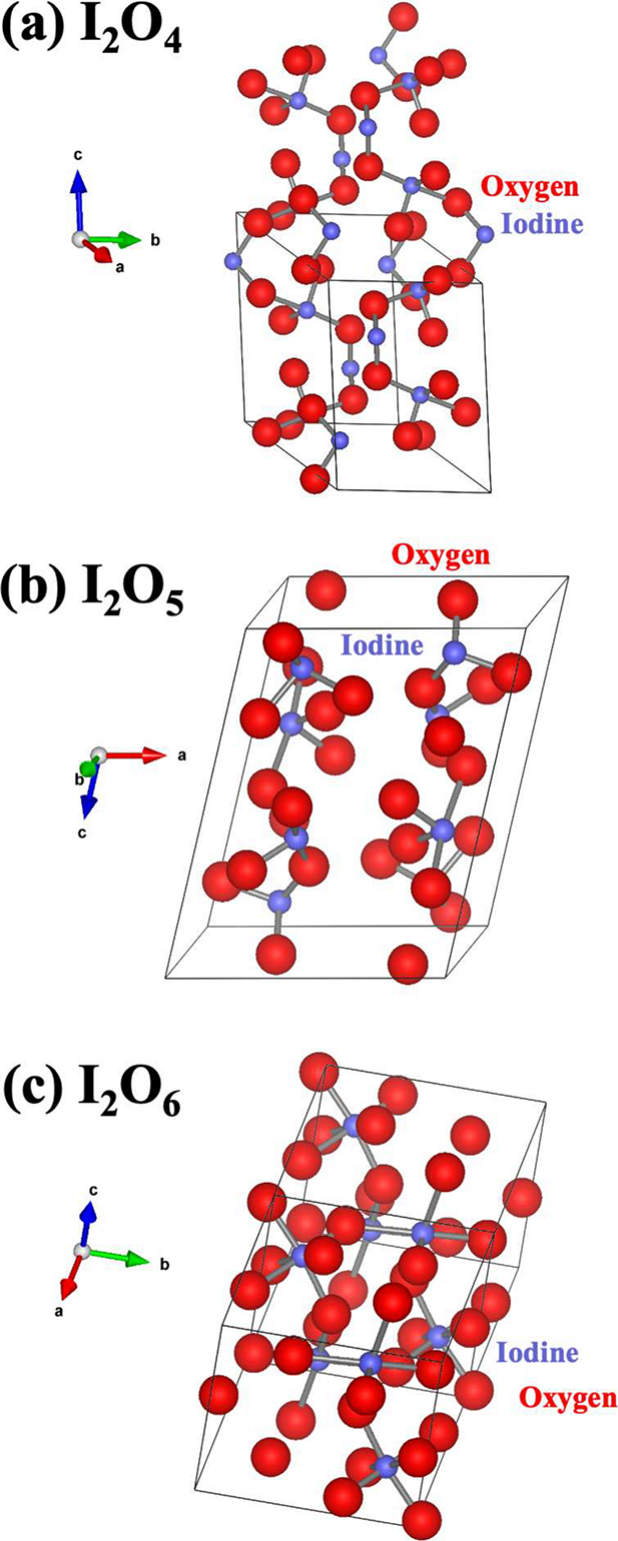
Crystal structures of I_2_O_*y*_ (*y* = 4, 5, and 6): (a) I_2_O_4_ (monoclinic, *P*2_1_/*c*, *Z* = 4), (b) I_2_O_5_ (monoclinic, *P*2_1_/*c*, *Z* = 4), and (c) I_2_O_6_ (triclinic, *P*1̅, *Z* = 2) crystals. The unit cells are outlined by solid black lines, and the molecular units, I_2_O_4_, I_2_O_5_ and I_4_O_12_, are identified by atoms denoted by solid circles.

Because of the larger size and more polarizable orbitals of the I atom than those of the O atom, the binary systems I_2_O_*y*_ accommodate increasing oxygen content structurally via adjustments of coordination numbers of I atoms and I–O–I bond angles but always stay between molecular- and framework-type crystal structures. Figures 1a–c illustrate the molecular units in I_2_O_4_ (monoclinic, *P*2_1_/*c*, *Z* = 4), I_2_O_5_ (monoclinic, *P*2_1_/*c*, *Z* = 4), and I_2_O_6_ (triclinic, *P*1̅, *Z* = 2) crystals. In all cases, neighboring molecules are connected by intermolecular I–O bonds (2.05–2.9 Å) that are significantly shorter than the van der Waals (vdW) distance (∼3.5 Å). Furthermore, each structure preferably features longer intermolecular I–O bonds along a crystallographic direction, i.e., the *b⃗*, *c⃗*, and *a⃗* directions for I_2_O_4_, I_2_O_5_, and I_2_O_6_, respectively. In Figures 1a–c, the molecular chain along the *c*-direction in I_2_O_4_, the layer-like substructure in the *ab*-plane in I_2_O_5_, and the framework configuration in I_2_O_6_ are noticeable. This, in turn, favors the formation of longer and weaker intermolecular I–O bonds along the *b⃗*, *c⃗*, and *a⃗* directions in I_2_O_4_, I_2_O_5_, and I_2_O_6_, respectively. H_2_O or SO_4_ acid molecules tend to enter the lattice, forming inserted layered structures to break up the IO_*x*_ framework, thereby rendering the I_2_O_*y*_ system thermodynamically unstable.

## Theoretical Computations

Computations were performed with the Vienna Ab initio Software Package (VASP)^26,27^ and the projector-augmented wave method^28^ for plane-wave density functional theory (DFT). We use the recently developed Strongly Constrained and Appropriately Normed (SCAN) exchange-correlation functional^29^ that obeys all known constraints on exact exchange correlation functionals for computing structural and dynamic properties of I_2_O_*y*_ crystals for comparison to experiments. We also benchmark this against DFT calculations using three semilocal exchange-correlation functionals, LDA,^30^ PBE^31^ and PBEsol^32^ as well as three hybrid exchange correlation functionals, B3LYP,^33^ HSE06,^34^ and PBE0.^35^

Structural optimization was carried out using the damped variable-cell-shape molecular dynamics (MD) scheme^36^ and conjugate gradient relaxation. The final optimization introduced as much as 5%–8% corrections for the lattice constants *b*, *c*, and *a* in I_2_O_4_, I_2_O_5_, and I_2_O_6_, respectively, and about one-third of these values for the other lattice constants. While DFT with LDA is a useful and powerful method, it has serious limitations. For example, it does not include long-range vdW interactions. In metals and semiconductors, this is not a serious problem; however, for molecular crystals that include highly polarizable ions such as O^2–^, I^–^, etc., there are large discrepancies in lattice constants of molecular solids and interatomic bond lengths. I_2_O_4_, I_2_O_5_, and I_2_O_6_ pose a serious difficulty in this context. Most computer programs have an average level of vdW interaction included, in addition to the LDA. With the average value of the vdW interaction parameter, the computed lattice constants for I_2_O_4_, I_2_O_5_, and I_2_O_6_ are larger, compared to the experimental values, and interatomic bond lengths are also longer. The value of the vdW interaction parameter (*s*_6_) had to be increased to obtain lattice constants that were close to experimental values. This is essential to accomplish prior to the PDOS computation. Specifically, we had to modify the global scaling factor from its nominal value of *s*_6_ = 0.75 to *s*_6_ =1.0 and 2.0 for I_2_O_5_ and I_2_O_6_, respectively, in order to reproduce experimental unit-cell volumes. This anisotropy results from the tight molecular netting in the *ac*, *ab*, and *bc* planes in the I_2_O_4_, I_2_O_5_, and I_2_O_6_ structures, respectively (see Figures 1a–c). Due to the large difference in the atomic size between I and O, the vdW interactions have a nontrivial impact on the calculated bond lengths and bond angles. In general, intermolecular bond lengths agree with experimental values, but intramolecular bond lengths are overestimated by 2%–3% (up to ∼5% in some cases).

**Figure 2:**
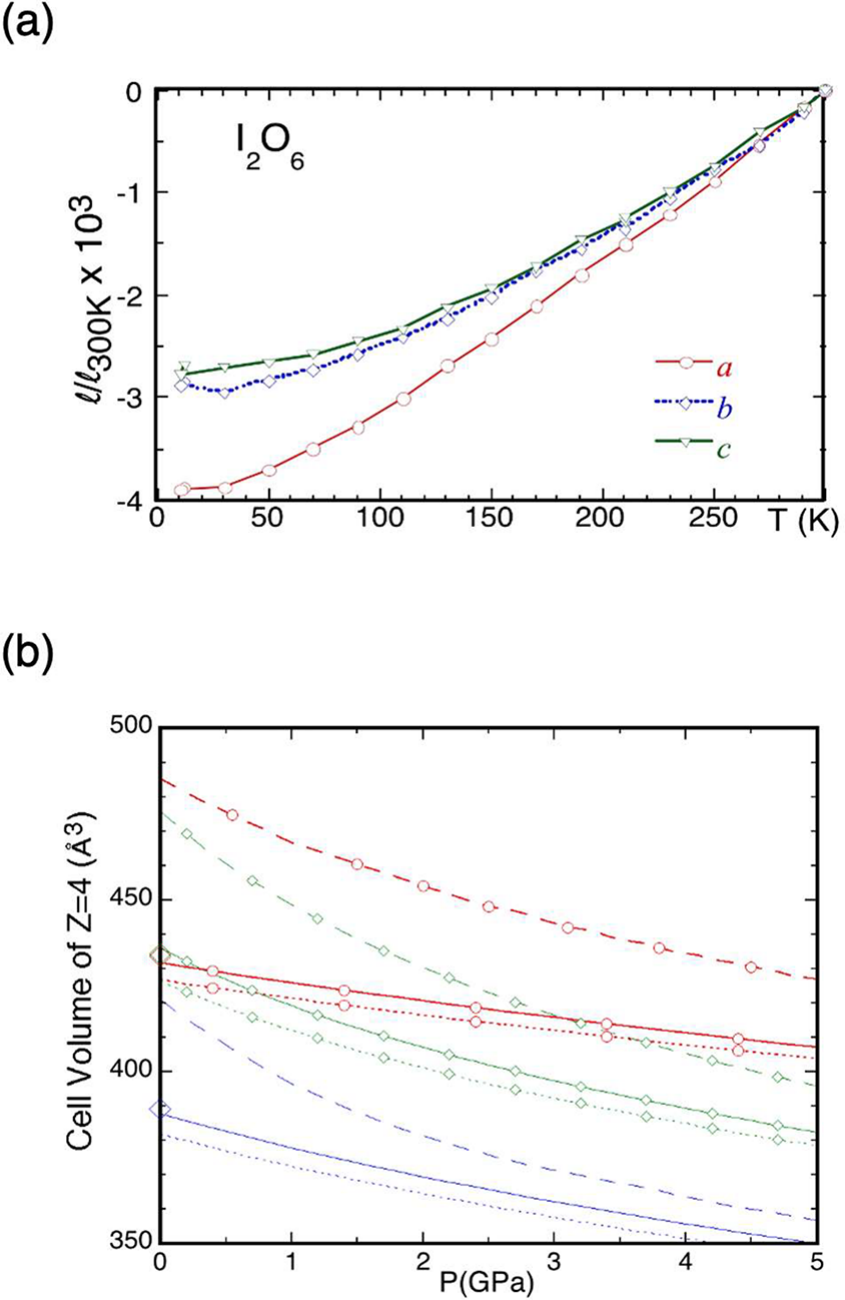
(a) Measured change in the lattice parameters of I_2_O_6_, relative to those at 300 K, reflecting the anisotropy in molecular bonding along the *ab*, *bc*, and *ac* planes. (b) Calculated equations of state for I_2_O_4_ (blue lines), I_2_O_5_ (green lines affixed with diamonds), and I_2_O_6_ (red lines affixed with circles). For each I_2_O_*y*_ member, we compare calculations without vdW force corrections (dashed lines), with vdW force correction but not including thermal contribution from phonons (dotted lines), and with both vdW force correction and phonon contributions at 300 K (solid lines). Experimental cell volumes for four formula units at ambient pressure are shown by large diamonds along the *P* = 0 axis.

The anisotropy in the bonding networks along the *ac*, *ab*, and *bc* planes in the I_2_O_4_, I_2_O_5_ and I_2_O_6_ structures is corroborated by experiments, through the anisotropic thermal expansion of the I_2_O_6_ lattice favoring the *a⃗* direction, as observed by neutron diffraction (Figure 2a). We calculated the EOS, thermal expansion, heat capacity, adiabatic bulk modulus, and thermal Grüneisen parameters (the weighted average of the mode Grüneisen parameters) for I_2_O_4_, I_2_O_5_, and I_2_O_6_. The calculated EOS in Figure 2b shows a substantial overestimation without the vdW correction and the adjustment of the *s*_6_ parameter. We find significant improvement in the EOS by including the thermal contribution of phonons in the quasi-harmonic approximation. The contribution to the equilibrium volume by thermal vibrations (including the zero-point motion), as a function of pressure, is ∼0.9%–2.2%. Interestingly, these values are comparable to those for MgO and Mg_2_SiO_4_,^37^ despite the large difference in the bulk modulii of 3D framework crystals of MgO and Mg_2_SiO_4_ (∼170 GPa) and iodine oxides (∼30 GPa). The three iodine oxides have a similar thermal pressure gradient of ∼1.7 GPa/1000 K.

A quantitative comparison of the combined structural and dynamic data with first-principles calculations permits a systematic assessment of the interplay between atomic and molecular forces and structural/thermodynamic properties. The phonon calculation was performed by the diagonalization of the dynamical matrices first computed on a 2 × 2 × 2 and subsequently interpolated to a 12 × 12 × 12 mesh in the first Brillouin zone using density functional perturbation theory^38^ and the visualization of the lattice-, intermolecular-, and intramolecular vibrational modes by the VESTA 3 software.^39^ INS, on the other hand, measures the neutron-weighted (NW) PDOS, according to
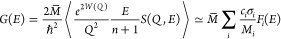
3where *c*_*i*_, σ_*i*_, *M*_*i*_, and *F*_*i*_(*E*) are the concentration, scattering cross section, mass and partial PDOS, respectively, for the *i*th atomic species. *M̅* is the mean sample mass, *n* is the Bose population factor, and *S*(*Q*, *E*) is the observed neutron scattering function for one-phonon excitations (after correction for multiphonon processes) as a function of energy *E* and wavevector *Q*. Corrections for the Debye–Waller factor, *e*^–2*W*(*Q*)^ = exp(−*u*^2^Q^2^), were made by using calculated *F*_*i*_(*E*). Here, ⟨···⟩ denotes an average over a wide range of observed *Q* values. The experimental NWPDOS was compared with that obtained from eq 3 using *F*_*i*_(*E*) from first-principles MD simulations convoluted with the instrumental resolution function.

**Figure 3:**
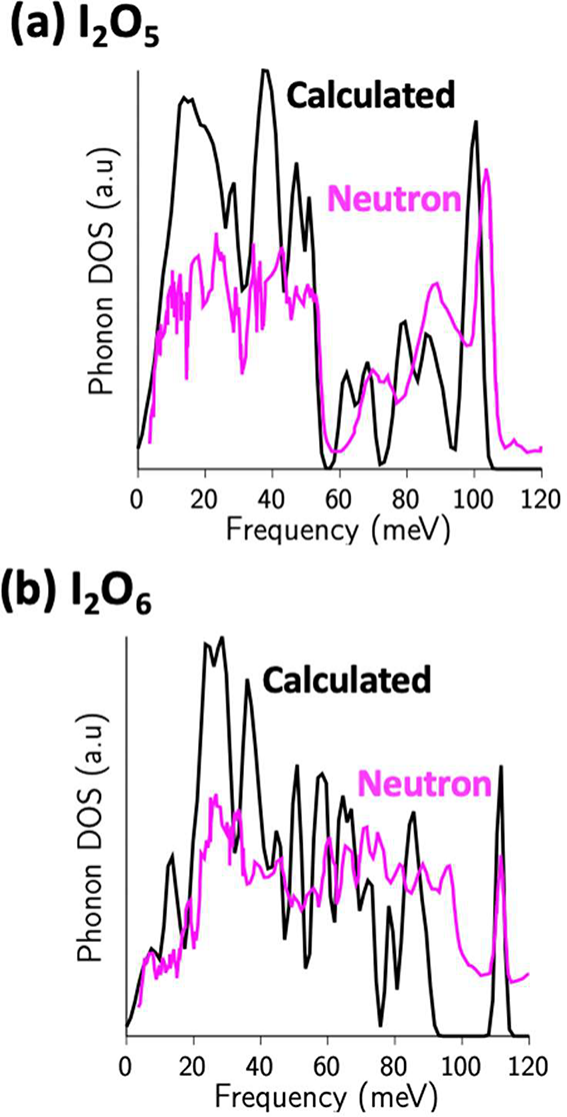
Experimentally measured and calculated neutron-weighted phonon densities of states for (a) I_2_O_5_ and (b) I_2_O_6_ normalized to the same integrated area.

The calculated PDOS and measured NWPDOS for I_2_O_5_ and I_2_O_6_ are shown in Figure 3. Lattice modes below ∼15 meV extend to the internal molecular vibrations of higher energies without obvious division, reflecting the comparable bond strengths connecting the atoms within the I_2_O_4_, I_2_O_5_, and I_2_O_6_ units and the atoms between neighbor molecules. For I_2_O_4_ (not shown) and I_2_O_5_, there is an ∼5 meV wide gap at ∼60 meV dividing the bending/rocking modes and the stretching vibrations; however, for I_2_O_6_, such a gap does not exist. Moreover, the high-energy one-phonon cutoff is observed at 104, 109, and 117 meV for I_2_O_4_, I_2_O_5_, and I_2_O_6_, respectively. These observations are consistent with the stiffening of the lattice by the stronger framework-like connection of molecular units with increasing oxygen content. Raman and IR zone-center modes agree well with the neutron peak positions, and the calculated PDOS shows some differences; e.g., for I_2_O_5_, the calculation predicts higher upper frequencies of molecular bending and stretching modes than observed values. Both stretching and bending modes are sensitive, respectively, to the overestimated intramolecular and underestimated intermolecular I–O bond lengths in SCAN-functional-based DFT simulations. The calculated PDOS and the Raman and IR frequencies show that this happens also in the case of I_2_O_4_.^40^ In the case of I_2_O_6_, the calculated I–O bond-stretching frequencies are clearly underestimated but the framework-like crystal structure apparently hardens the bending modes, thereby filling the gaps seen in I_2_O_4_ and I_2_O_5_. Finally, the observed phonon spectra are not as sharp as the calculated ones, which is likely due to residual multiphonon and small-size crystallite broadening effects that are not accounted for in the DFT simulations.

The simulations account for the crystal structures as well as the lattice dynamics of I_2_O_*y*_ systems reasonably well, the 10–15% energy shifts of high-energy modes notwithstanding. This is caused by the compromise between intermolecular and intramolecular interactions due to the vdW correction.

**Figure 4:**
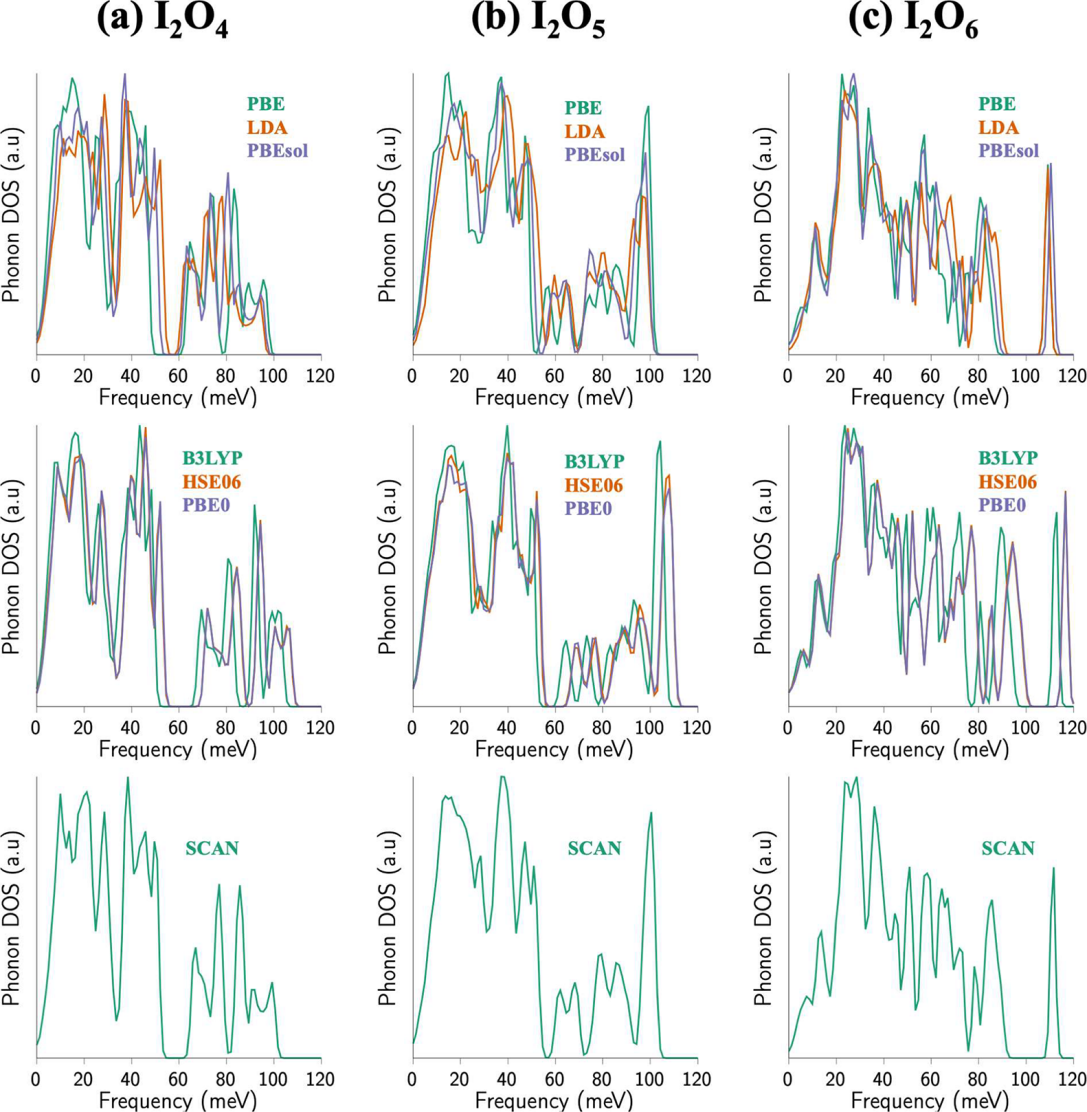
Phonon densities of states for (a) I_2_O_4_, (b) I_2_O_5_ and (c) I_2_O_6_ computed using three semilocal functionals, PBE, LDA and PBEsol (top row), three hybrid functionals, B3LYP, HSE06, and PBE0 (middle row), and a meta functional, SCAN (bottom row). The overestimated bond lengths in the PBE functional result in softer phonon vibrational frequencies, which is corrected in the PBEsol, LDA and SCAN xc functionals.

Figure 4 shows the PDOS for (a) I_2_O_4_, (b) I_2_O_5_, and (c) I_2_O_6_ crystals computed using multiple exchange correlation functionals. Semilocal functionals like PBE overestimate I–O intramolecular bond lengths, resulting in a mechanically soft crystal and lower vibrational frequencies. Other semilocal exchange correlation functions, including LDA, PBEsol, as well as hybrid functionals like B3LYP, HSE06, and PBE, as well as the meta-functional, SCAN, can reproduce accurate crystal structures and bond lengths and, therefore, result in higher and experimentally accurate vibrational frequencies.

**Figure 5:**
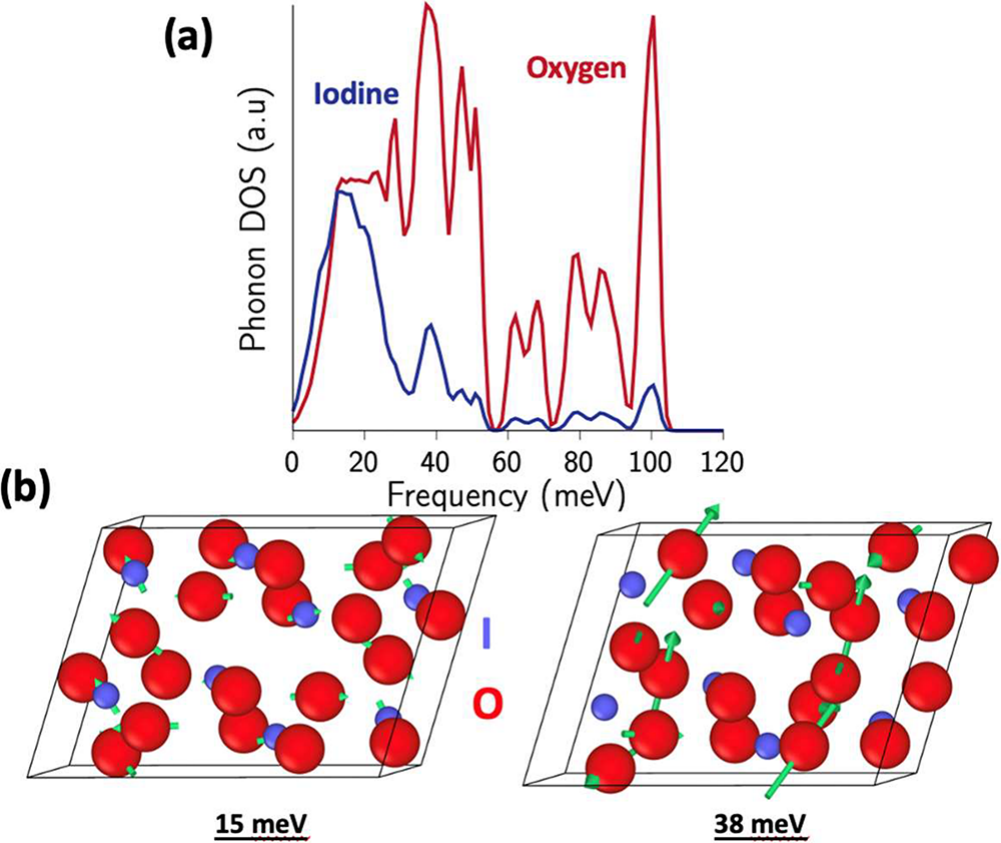
Calculated phonon densities of states for (a) I_2_O_5_ decomposed by atom type. Low-frequency (high-frequency) modes are characterized by vibrations of primarily I (O) atoms. (b) Atomic displacements (in green) corresponding to eigenvectors of these modes in I_2_O_5_ reflect this atom decomposition.

Figure 5 shows the atom-decomposed computed PDOS for I_2_O_5_, along with the eigenvectors for selected high-intensity states at the Gamma point. Low-frequency modes up to 40 meV are dominated by the displacement and motion of the heavier I atoms (e.g., mode at 15 meV in Figure 5b), while high-energy modes in the range of 40–120 meV are characterized by the displacements of lighter O atoms (e.g., mode at 38 meV in Figure 5b).

**Figure 6:**
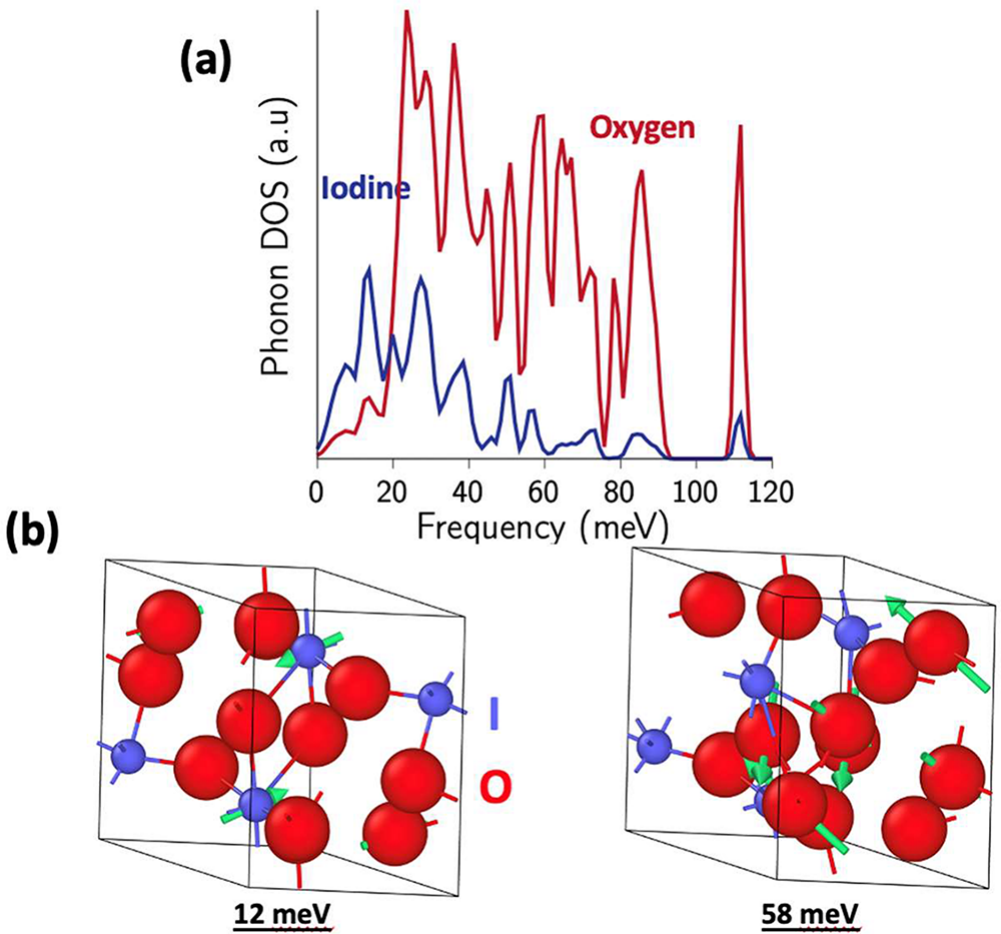
Calculated phonon densities of states for (a) I_2_O_6_ decomposed by atom type. Low-frequency (high-frequency) modes are characterized by vibrations of primarily I (O) atoms. (b) Atomic displacements (in green) corresponding to eigenvectors of these modes in I_2_O_6_ reflect this decomposition.

Figure 6 shows the atom-decomposed computed PDOS for I_2_O_6_, along with the eigenvectors for selected high-intensity states at the Gamma point. Low-frequency modes up to 40 meV are dominated by the displacement and motion of heavier I atoms (e.g., mode at 12 meV in Figure 6b), while high-energy-frequency modes in the range of 40–120 meV are characterized by the displacements of lighter O atoms (e.g., mode at 58 meV in Figure 5b).

In summary, neutron measurements of the crystal structures and phonon densities of states of newly prepared, pure, anhydrous I_2_O_5_ and I_2_O_6_ samples provide crucial experimental data for the optimization of first-principles MD simulations of the structures and dynamics of I_2_O_4_, I_2_O_5_, and I_2_O_6_. Important thermodynamic properties were computed to further our understanding of these materials under extreme conditions.

## Supporting Information Available

Computed lattice constants of I_2_O_4_, and I_2_O_5_, I_2_O_6_crystals, using different exchange correlations functionals are given, along with illustrations of the molecules (PDF)
