# Supplementary material for: Inelastic Neutron Scattering Study of Phonon Density of States of Iodine Oxides and First-Principles Calculations
Source: J Phys Chem Lett. 2023 Nov 2;14(44):10080–7. doi: 10.1021/acs.jpclett.3c02357 (PMC10641886; doi:10.1021/acs.jpclett.3c02357)
Supplement: Supplementary file 1 — jz3c02357_si_001.pdf [file jz3c02357_si_001.pdf]

Supporting Information

Inelastic Neutron Scattering Study of Phonon Density of States of Iodine Oxides and First-Principles Calculations

Alexander I. Kolesnikov,[1] Aravind Krishnamoorthy,[2] Ken-ichi Nomura,[3] Zhongqing Wu,[4] Douglas L. Abernathy,[1] Ashfia Huq,[5] Garrett E. Granroth,[1] Karl O. Christe,[6] Ralf Haiges,[6] Rajiv K. Kalia,[3] Aiichiro Nakano,[3] and Priya Vashishta[3]

[1]*Neutron Scattering Division, Oak Ridge National Laboratory, Oak Ridge, TN 37831-6473, USA*

[2]*J. Mike Walker '66 Department of Mechanical Engineering, Texas A&M University, College Station, TX 77843, USA*

[3]*Collaboratory for Advanced Computing and Simulations, Department of Chemical Engineering & Materials Science, Department of Physics & Astronomy, and Department of Computer Science, University of Southern California, Los Angeles, CA 90089, USA*

[4]*School of Earth and Space Sciences, University of Science and Technology of China, Hefei, Anhui 230026, China*

[5]*Sandia National Laboratories, Livermore, California 94551, United States*

[6]*Loker Research Institute and Department of Chemistry, University of Southern California, Los Angeles, CA 90089, USA*

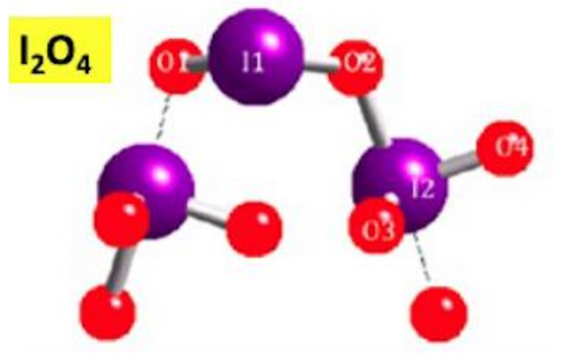

Figure S1: Structure of I_2O_4 molecule.

TABLE S1: Computed lattice constants of I_2O_4 crystal using different exchange correlations functionals.

	**I_2O_4**					
	a	*b*	*c*	*α*	*β*	*γ*
**LDA**	7.298	6.611	7.896	90.0	115.1	90.0
**GGA – PBE**	8.044	7.046	8.360	90.0	115.8	90.0
**PBEsol**	7.661	6.823	8.088	90.0	115.6	90.0
**SCAN**	7.739	7.739	8.210	90.0	115.9	90.0
**PBE0**	7.969	6.895	8.295	90.0	116.0	90.0
**B3LYP**	8.178	7.047	8.449	90.0	116.2	90.0
**HSE06**	7.986	6.900	8.306	90.0	116.1	90.0

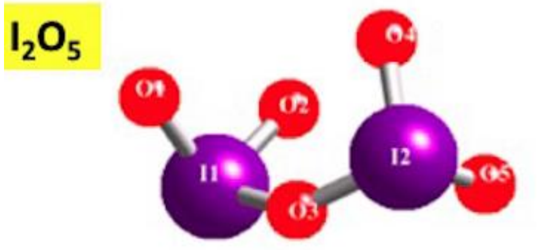

Figure S2: Structure of I_2O_5 molecule.

TABLE S2: Computed lattice constants of I_2O_5 crystal using different exchange correlations functionals.

	**I_2O_5**					
	a	*b*	*c*	*α*	*β*	*γ*
**LDA**	7.786	4.901	10.697	90.0	107.8	90.0
**GGA – PBE**	8.474	5.207	11.167	90.0	107.3	90.0
**PBEsol**	8.055	5.042	10.865	90.0	107.6	90.0
**SCAN**	8.157	5.030	10.931	90.0	107.3	90.0
**PBE0**	8.333	5.096	10.967	90.0	107.0	90.0
**B3LYP**	8.481	5.224	11.195	90.0	107.0	90.0
**HSE06**	8.411	5.111	10.974	90.0	106.8	90.0

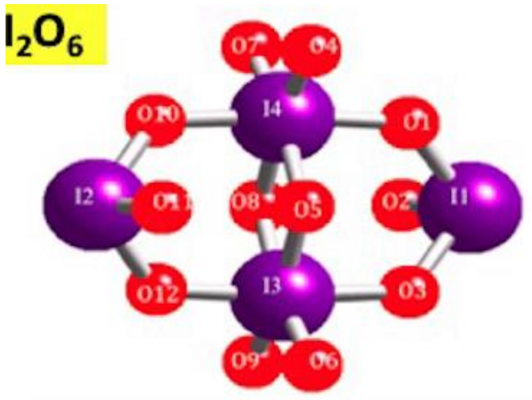

Figure S3: Structure of I_2O_6 molecule.

TABLE S3: Computed lattice constants of I_2O_6 crystal using different exchange correlations functionals.

	**I_2O_6**					
	a	*b*	*c*	α	β	γ
**LDA**	4.989	6.655	6.706	97.9	96.3	107.6
**GGA – PBE**	5.450	6.861	6.893	98.4	96.8	107.6
**PBEsol**	5.400	6.754	6.794	98.3	96.4	107.5
**SCAN**	5.200	6.738	6.791	98.1	96.1	107.0
**PBE0**	5.393	6.723	6.765	98.2	96.1	107.0
**B3LYP**	5.462	6.850	6.900	98.3	96.0	107.0
**HSE06**	5.395	6.731	6.777	97.8	96.2	107.0
